# Perioperative Predictors and Temporal Trajectories of Postoperative Cognitive Decline After Carotid Endarterectomy: A Prospective Cohort Study

**DOI:** 10.3390/brainsci16101032

**Published:** 2026-09-27

**Authors:** Ziyuan Shen, Jingshu Hong, Zhongxiong Yao, Xiaoxiao Wang, Ming Liu, Yang Zhou, Taotao Liu, Xiangyang Guo, Zhengqian Li

**Affiliations:** 1 Department of Anesthesiology, Peking University Third Hospital, No. 49 North Garden Road, Haidian District, Beijing 100191, China; 2411110576@bjmu.edu.cn (Z.S.); hongjingshu@bjmu.edu.cn (J.H.); 2511210401@bjmu.edu.cn (Z.Y.); xiao910129@bjmu.edu.cn (X.W.); liuming@fjsfy.com (M.L.); zhouyang@pku.edu.cn (Y.Z.); liutaotao1101@pku.edu.cn (T.L.); 2 Executive Office, Beijing Center of Quality Control and Improvement on Clinical Anesthesia, Beijing 100191, China; 3 Research Center of Clinical Epidemiology, Peking University Third Hospital, Beijing 100191, China; 4 Key Laboratory of Epidemiology of Major Diseases (Peking University), Ministry of Education, Beijing 100191, China; 5 Fujian Maternity and Child Health Hospital, College of Clinical Medicine for Obstetrics & Gynecology and Pediatrics, Fujian Medical University, Fuzhou 350001, China; 6 State Key Laboratory of Vascular Homeostasis and Remodeling, Department of Anesthesiology, Peking University Third Hospital, Beijing 100191, China

**Keywords:** carotid artery stenosis, carotid endarterectomy, cognitive function, risk factor

## Abstract

**Highlights:**

**What are the main findings?**

- Five perioperative factors were independently associated with early postoperative cognitive decline after carotid endarterectomy: preoperative triglyceride level, preoperative cerebral hypoperfusion volume, surgical laterality, intraoperative mean arterial pressure decline, and visual analogue scale on postoperative day 1.
- Four distinct cognitive trajectory subtypes were identified among patients with complete 30-day follow-up, revealing heterogeneous patterns including stable, transient, delayed, and persistent decline.

**What are the implications of the main findings?**

- A perioperative risk model integrating metabolic, cerebral perfusion, surgical, hemodynamic, and pain-related variables may help identify patients at high risk of cognitive decline after carotid endarterectomy, supporting individualized preoperative risk assessment.

**Abstract:**

Background/Objectives: Carotid artery stenosis is a well-established risk factor for vascular cognitive impairment. Although carotid endarterectomy (CEA) reduces the risk of ischemic stroke, its effect on cognitive function remains uncertain. Most previous studies have relied on mean changes in cognitive scores and have not defined postoperative cognitive decline using patient-level thresholds for the Montreal Cognitive Assessment (MoCA). Moreover, few studies have integrated perioperative variables across baseline, intraoperative, and postoperative domains to examine heterogeneity in cognitive outcomes. We therefore defined postoperative cognitive decline using threshold-based MoCA changes and prospectively evaluated perioperative factors associated with distinct cognitive outcomes after CEA. Methods: We prospectively enrolled older patients undergoing elective CEA under general anesthesia. Of 183 patients screened, 162 were ultimately enrolled and analyzed. All patients were managed according to standardized anesthetic and surgical protocols. Preoperative clinical, laboratory, and imaging variables, intraoperative hemodynamic parameters, and postoperative clinical indicators were collected. The primary outcome was cognitive decline at discharge, defined as a decrease of ≥2 points from baseline in the MoCA score. The secondary outcome was cognitive decline at 30 days, defined as a ≥3-point decrease in the telephone MoCA (t-MoCA) score relative to the corresponding baseline domains. Results: Among the 162 enrolled patients, preoperative triglyceride level, preoperative cerebral hypoperfusion volume, surgical laterality, intraoperative mean arterial pressure (MAP) decline, and visual analogue scale (VAS) on postoperative day 1 were independently associated with early postoperative cognitive decline. A prediction model incorporating these five factors achieved an area under the curve (AUC) of 0.752 (95% CI: 0.670–0.835) for cognitive decline before discharge. For 30-day cognitive decline, the model demonstrated an AUC of 0.717 (95% CI: 0.606–0.828). All patients with complete 30-day follow-up were stratified into four cognitive trajectory subtypes, and their longitudinal cognitive changes were visualized using a Sankey diagram. Conclusions: A perioperative model incorporating metabolic, cerebral perfusion, surgical, hemodynamic, and pain-related variables may help identify patients at increased risk of cognitive decline after CEA. The trajectory analysis further reveals heterogeneous postoperative cognitive patterns, including transient, delayed, and persistent decline. These findings support individualized perioperative risk assessment and structured cognitive follow-up. However, no internal or external validation was performed, and further testing is required in larger multicenter cohorts.

## 1. Introduction

Carotid artery stenosis is an established risk factor for vascular cognitive impairment. Cognitive consequences may range from mild cognitive impairment to vascular dementia and may coexist with neurodegenerative disorders such as Alzheimer’s disease [1]. Even low-grade carotid stenosis, despite its relatively low stroke risk, has been associated with progressive brain atrophy and cognitive decline. Older patients with mild carotid stenosis may experience faster deterioration in memory and executive function than those without carotid stenosis [2].

CEA is a major treatment for carotid stenosis and can reduce the risk of ischemic stroke, but its effect on cognition remains controversial [3,4]. Preoperative cognitive decline, cerebral small vessel disease, intraoperative hypoperfusion or microembolization, and postoperative hyperperfusion may all influence postoperative cognitive outcomes. Previous research has indicated that in patients with carotid stenosis complicated by cerebral small vessel disease, cerebral blood flow increased postoperatively in 23% of patients, whereas cognitive function improved in only 11% [4]. Some patients may improve after restoration of cerebral perfusion, whereas others may develop new or worsened cognitive decline. Previous studies on perioperative cognitive function following CEA have predominantly relied on group-averaged cognitive score alterations [5,6]. However, aggregated mean values may conceal clinically relevant interindividual heterogeneity, since cognitive gains in a subset of patients can statistically counterbalance overt cognitive deterioration in others. Accordingly, individualized, threshold-based definitions are indispensable for rigorous clinical evaluation and decision-making. In addition, practice effect associated with repeated cognitive assessments, along with other confounding factors such as anxiety, sleep quality, intraoperative blood pressure fluctuations, and early postoperative status, is not always considered together. These limitations highlight the need for prospective studies that evaluate individual cognitive changes using multidimensional perioperative data.

In this prospective cohort study, cognitive function was assessed before surgery, at discharge, and 30 days after surgery. Postoperative cognitive decline was defined using threshold-based reductions in MoCA or t-MoCA scores relative to baseline. We aimed to identify perioperative factors associated with early cognitive decline after CEA and to characterize cognitive outcome trajectories from discharge to 30 days.

## 2. Materials and Methods

### 2.1. Participants

This study was approved by the Ethics Committee of Peking University Third Hospital (approval number: M20250534, approved on 3 June 2025) and was registered with the Chinese Clinical Trial Registry (registration number: ChiCTR2500104090, registered on 10 June 2025). All patients were enrolled after the study was registered. The study was conducted in strict accordance with the Declaration of Helsinki. We prospectively enrolled patients scheduled for elective CEA under general anesthesia in the Department of Neurosurgery at Peking University Third Hospital between 2025 and 2026. All patients provided written informed consent.

Inclusion criteria were age ≥ 60 years; American Society of Anesthesiologists (ASA) physical status classification I-III; scheduled for elective carotid endarterectomy under general anesthesia; and not concurrently participating in other clinical studies. Exclusion criteria were preoperative dementia or cognitive impairment attributed to other conditions, such as traumatic brain injury or neurodegenerative diseases; severe visual or auditory impairment, language disorders, and/or communication difficulties; abnormal activities of daily living (ADL) scores; alcohol or substance dependence; and concurrent severe cardiac, hepatic, renal, or hematological diseases, as well as a history of delirium or other psychiatric disorders.

### 2.2. Perioperative Anesthesia Management Protocol

All patients received general anesthesia with endotracheal intubation. Upon arrival in the operating room, standard monitoring was applied, including electrocardiography, non-invasive blood pressure, pulse oximetry, and regional cerebral oxygen saturation. A radial arterial catheter was inserted under local anesthesia for continuous invasive arterial blood pressure monitoring. For anesthesia induction, after adequate preoxygenation and denitrogenation, sufentanil 0.2–0.3 μg/kg, etomidate 0.2 mg/kg, lidocaine 1–1.5 mg/kg, and rocuronium 0.6 mg/kg were administered intravenously sequentially, with propofol (total dose approximately 1 mg/kg) given in divided doses according to the patient’s hemodynamic status. If hypotension occurred after induction, ephedrine (5–10 mg), norepinephrine (4–8 μg), or phenylephrine (40 μg) was administered intravenously as needed. Anesthesia was maintained with a combination of intravenous and inhalational agents, using sevoflurane (0.7 MAC) and a continuous infusion of remifentanil (0.05–0.1 μg/kg/min). During carotid artery clamping, all patients received 5000 IU of heparin intravenously. Ephedrine, norepinephrine, or phenylephrine was administered as appropriate to increase blood pressure to 20% above baseline, with subsequent dynamic adjustments guided by cerebral oxygen saturation to maintain cerebral perfusion pressure. No shunt was used in any patient. After unclamping of the carotid artery, blood pressure was reduced to 20% below baseline; urapidil or nicardipine was administered intravenously as needed. Indocyanine green angiography was performed to assess vascular patency and suture integrity. At the end of surgery, a single intravenous dose of flurbiprofen axetil or sufentanil was given for analgesia, and ondansetron was administered to prevent postoperative nausea and vomiting. After extubation, patients were observed in the operating room or post-anesthesia care unit for at least 30 min and were returned to the ward only after vital signs had stabilized and no specific discomfort was observed.

### 2.3. Data Collection

#### 2.3.1. Preoperative Data Collection

Complete preoperative clinical, laboratory, and imaging data were collected for all enrolled patients. Demographic variables included age, sex, body mass index (BMI), smoking history, alcohol consumption history, dominant hand, educational level, regular physical exercise, and leisure activity participation. Preoperative comorbidities, including hypertension, coronary artery disease, diabetes, cerebral infarction, and respiratory dysfunction, were documented. Preoperative brain imaging metrics included hypoperfusion volume and infarct core volume derived from preoperative cerebral computed tomography perfusion (CTP). The clinicians interpreting CTP images were blinded to patient clinical information. Laboratory parameters included white blood cell count, red blood cell count, hemoglobin, and lipid profiles. All blood samples were collected after an overnight fast. Preoperative baseline mean arterial pressure (MAP), Pittsburgh Sleep Quality Index (PSQI), Self-Rating Anxiety Scale (SAS), and baseline MoCA score were also recorded.

#### 2.3.2. Anesthesia and Surgery-Related Data

Intraoperative anesthesia and surgical data were collected, including surgical laterality, the magnitude of decline in regional cerebral oxygen saturation (rSO2) on the operative side, carotid artery clamping time, the maximum increase in mean arterial pressure relative to baseline, the maximum decrease in mean arterial pressure relative to baseline, the overall maximum fluctuation range of MAP during surgery, and total anesthesia time. MAP was continuously and real-time monitored using an invasive arterial catheter throughout surgery.

#### 2.3.3. Postoperative Data

Postoperative delirium was assessed using the 3-Minute Diagnostic Interview for Confusion Assessment Method-Defined Delirium (3D-CAM), a standardized diagnostic interview instrument. The CAM algorithm comprises four core diagnostic features: (1) acute onset and fluctuating course, (2) inattention, (3) disorganized thinking, and (4) altered level of consciousness. A diagnosis of delirium requires the presence of both features 1 and 2, plus either feature 3 or 4. The 3D-CAM is a standardized, abbreviated tool modified from the CAM diagnostic algorithm, consisting of 22 standardized questions and observational items, with diagnostic thresholds identical to those of the CAM. It is suitable for rapid perioperative screening of postoperative delirium in elderly patients. In this study, geriatricians blinded to patients’ clinical data evaluated the occurrence of postoperative delirium twice daily, at 08:00–10:00 and 14:00–16:00, on postoperative days 1 through 5.

Postoperative pain was assessed using the visual analogue scale (VAS). Trained assessors recorded pain scores from postoperative day 1 to day 5. Participants marked their pain intensity on a 0–10 cm scale, where 0 indicated no pain and 10 indicated intolerable pain.

#### 2.3.4. Cognitive Function Assessment

Consistent with the methodology reported by Kennedy et al. [7], we assessed patients’ cognitive function using MoCA at baseline and before discharge, while the t-MoCA was adopted for cognitive reassessment at postoperative 30 days.

The primary outcome was cognitive decline at discharge. All enrolled patients underwent MoCA assessment before surgery and at discharge. The MoCA is a standardized screening tool that evaluates visuospatial/executive function, naming, memory, attention, language, abstraction, delayed recall, and orientation, with a maximum score of 30 points. Based on previous literature, a MoCA increase of ≥2 points was defined as cognitive improvement, whereas a decrease of ≥2 points was defined as cognitive decline [8,9].

The secondary outcome was cognitive decline at 30 days postoperatively. Cognitive function at this time point was reassessed using the Telephone Montreal Cognitive Assessment (t-MoCA). The t-MoCA is adapted from the standard MoCA by omitting the visuospatial/executive and naming domains, resulting in a maximum score of 22 points. For comparison with baseline, the corresponding visuospatial/executive and naming subscales were omitted from the baseline MoCA score. An increase of ≥3 points in the t-MoCA score was defined as cognitive improvement, whereas a decrease of ≥3 points was defined as cognitive decline [7].

For patients who completed the 30-day follow-up, cognitive outcome trajectories were classified into four subtypes based on the combination of cognitive change at discharge and at 30 days:

(1) Stable cognition: A MoCA decrease of <2 points at discharge and a t-MoCA decrease of <3 points at 30 days;
(2) Transient cognitive decline: A MoCA decrease of ≥2 points at discharge and a t-MoCA decrease of <3 points at 30 days;
(3) Delayed cognitive decline: A MoCA decrease of <2 points at discharge and a t-MoCA decrease of ≥3 points at 30 days;
(4) Persistent cognitive decline: A MoCA decrease of ≥2 points at discharge and a t-MoCA decrease of ≥3 points at 30 days.

To control the practice effect induced by repeated testing, validated parallel versions of the MoCA were adopted at three assessment time points (preoperatively, before discharge, and 30 days after surgery). All cognitive assessments were performed by a single physician with official MoCA certification, who was fully blinded to the patients’ intraoperative parameters, surgical laterality, and laboratory findings. All assessments were conducted in a quiet, private room at the same time of day.

### 2.4. Sample Size Calculation

The sample size was estimated based on previous CEA studies and modeling requirements. Prior studies have identified approximately 3–4 factors associated with cognitive decline after CEA, with an estimated incidence of postoperative cognitive decline of 20.5% [10]. Using an events-per-variable ratio of 10 for model development, the minimum required sample size was estimated to be 146 patients. After allowing for an anticipated 10% attrition rate, the target sample size was set at 162 patients.

### 2.5. Statistical Analysis

Statistical analyses were performed using IBM SPSS Statistics (version 26.0) and R software (version 4.5.3). A two-sided *p* value < 0.05 was considered statistically significant. Baseline characteristics were first compared between groups. For continuous variables, normality was assessed using the Shapiro–Wilk test. Normally distributed data were presented as mean ± standard deviation, and comparisons between two groups were performed using the independent-sample *t*-test, while comparisons among multiple groups were conducted using one-way analysis of variance (ANOVA) followed by least significant difference (LSD) post hoc tests. Non-normally distributed continuous variables were presented as median (interquartile range), with intergroup comparisons performed using the Mann–Whitney U test for two groups and the Kruskal–Wallis H test for multiple groups. Categorical variables, including both binary and multicategorical variables, were presented as frequencies (percentages) and compared using the chi-square test or Fisher’s exact test as appropriate. Missing data were handled using a complete-case analysis; patients with missing values in key variables were excluded from the analysis.

After baseline comparisons, candidate perioperative variables were entered into a least absolute shrinkage and selection operator (LASSO) regression model to reduce overfitting and multicollinearity (R package glmnet, version 4.5.3). The optimal penalty parameter λ was selected using 10-fold cross-validation. Variables with non-zero coefficients under the λ_min_ criterion were retained as candidate predictors. Coefficient path plots, cross-validation error plots, and coefficient bar plots were generated. To assess the stability of variable selection, we performed bootstrap resampling (1000 iterations). In each resampling, LASSO regression was re-run, and the selection frequency of each candidate variable was recorded. The candidate variables selected by LASSO were incorporated into a multivariable binary logistic regression model with backward elimination based on likelihood ratio statistics to calculate adjusted odds ratios (ORs) and 95% confidence intervals (CIs). Variables with *p* < 0.05 were considered independently associated with the outcome. Model discrimination was assessed using receiver operating characteristic (ROC) curves (Prism version 10.4.1), calibration using calibration plots (R package rms, version 4.5.3), and clinical utility using decision curve analysis (R package dcurves, version 4.5.3).

A Sankey diagram was used to visualize the cohort flow characteristics of cognitive outcome transitions from discharge to 30 days postoperatively in patients with complete follow-up (R package ggalluvial, version 4.5.3).

## 3. Results

A total of 183 patients scheduled for elective CEA under general anesthesia in the Department of Neurosurgery at Peking University Third Hospital between 2025 and 2026 were screened, and 178 met the inclusion criteria. During the study period, 5 patients developed postoperative stroke, transient ischemic attack (TIA), or cerebral hyperperfusion syndrome (CHS). Seven patients had missing data, and four had protocol deviations; therefore, 162 patients were included in the final analysis. At discharge (postoperative days 4 to 5; median 4 days; 4 days *n* = 150; 5 days *n* = 12), 28 patients (17.3%) showed cognitive improvement, with a maximum MoCA increase of 7 points; 60 patients (37.0%) showed cognitive decline, with a maximum MoCA decrease of 7 points; and 74 patients (45.7%) remained cognitively stable. A total of 119 patients completed the 30-day telephone follow-up. At 30 days, 14 patients (11.8%) showed cognitive improvement, 34 (28.6%) showed cognitive decline, and 71 (59.6%) remained stable (Figure 1). Of the 43 patients who were lost to follow-up, 20 were unreachable by telephone, 17 declined to participate in the cognitive assessment, and 6 were unable to complete the assessment because of hoarseness.

### 3.1. Factors Associated with Differential Cognitive Outcomes

Among the 162 patients, the median preoperative MoCA score was 24 (22–26), and the median MoCA score at discharge was 23 (21–26), corresponding to a median change of −1.0 (−2.0 to 1.0). To describe outcome heterogeneity, patients were classified into three groups: improved (*n* = 28), stable (*n* = 74), and declined (*n* = 60). In the improved group, the MoCA score increased by 2.5 (2.0, 3.0) points from baseline, whereas in the declined group, the MoCA score decreased by 3.0 (2.0, 4.0) points. Patients in the improved group were younger than those in the other two groups, and the proportion of left-handed patients was higher in this group. Detailed comparisons of perioperative variables are shown in Table 1.

### 3.2. Variable Selection for the Predictive Model

The primary regression analysis used a binary outcome (cognitive decline vs. no cognitive decline) to identify perioperative factors independently associated with the clinical event of cognitive decline. LASSO regression was applied to all candidate perioperative variables, including baseline MoCA. The λ value was selected according to the minimum criterion. Six variables were retained: preoperative triglyceride level, preoperative cerebral hypoperfusion volume, surgical laterality, maximum MAP decrease relative to baseline during surgery, postoperative delirium, and VAS on postoperative day 1 (Figure 2). Bootstrap resampling (1000 iterations) showed that all six retained variables had selection frequencies greater than 0.5, with the two strongest predictors being maximum MAP decrease relative to baseline during surgery and VAS on postoperative day 1. And postoperative delirium had a selection frequency of 0.531, which was only marginally above the critical threshold of 0.5. The detailed selection frequencies for all candidate variables are provided in Table S1. These variables were then included in a multivariable logistic regression model. Five variables remained independently associated with cognitive decline: preoperative triglyceride level, cerebral hypoperfusion volume, surgical laterality, maximum MAP decrease during surgery, and VAS on postoperative day 1 (Table 2). Given that change scores may be influenced by regression to the mean, and that patients with higher baseline cognitive function have greater room for decline, we performed a sensitivity analysis by forcing baseline MoCA into the multivariable logistic regression model. As shown in Table S2, after adjusting for baseline MoCA, preoperative triglyceride level, cerebral hypoperfusion volume, surgical laterality, maximum MAP decrease during surgery, and VAS on postoperative day 1 remained significantly associated with cognitive decline at discharge. In addition, baseline MoCA itself was independently associated with cognitive decline. These findings suggest that the perioperative risk factors identified in this study exert effects on cognitive decline independent of baseline cognitive level, and that regression to the mean cannot explain this association.

A prediction model for postoperative cognitive decline was constructed using the independent risk factors identified by multivariable logistic regression. The model equation was as follows: logit(P) = −4.053 + 0.613* Triglycerides + 0.008* Hypoperfusion Volume + 0.920* Side of Surgery (Left) + 0.047*(%ΔMAPbase − min) + 0.280* VAS on postoperative day 1. ROC curve analysis indicated good discrimination, with an AUC of 0.752 (95% CI: 0.670–0.835). The calibration curve showed agreement between predicted probabilities and observed event rates. Decision curve analysis suggested a positive net clinical benefit across a range of threshold probabilities. When this early prediction model was applied to the 30-day postoperative outcomes in the 119 patients available for follow-up, it maintained moderate discriminative ability, with an AUC of 0.717 (95% CI: 0.606–0.828) (Figure 3).

### 3.3. Differential Cognitive Outcomes at 30 Days Postoperatively

At 30 days after surgery, cognitive function was reassessed by telephone using the t-MoCA in 119 patients; 43 patients were lost to follow-up. Baseline demographic characteristics, intraoperative hemodynamic parameters, and perioperative clinical variables were compared between patients who completed follow-up and those lost to follow-up. No statistically significant differences were observed (Table S3).

A Sankey diagram was used to visualize cognitive transitions from discharge to 30 days after surgery in 119 patients (Figure 4). Patients with delayed cognitive decline did not meet the decline threshold at discharge but showed decline at 30 days, whereas those with transient cognitive decline declined at discharge but recovered by 30 days. Among the 46 patients with cognitive decline at discharge, 26 (56.5%) had persistent cognitive decline at 30 days; among the 73 patients without cognitive decline at discharge, 8 (11.0%) developed delayed cognitive decline.

## 4. Discussion

In this prospective cohort of 162 patients undergoing CEA, postoperative cognitive decline was defined using patient-level MoCA thresholds at discharge and 30 days. Five perioperative factors were independently associated with early cognitive decline: preoperative triglyceride level, cerebral hypoperfusion volume, surgical laterality, maximum intraoperative MAP decrease, and VAS on postoperative day 1. A model incorporating these variables showed acceptable discrimination for early cognitive decline and moderate discrimination for 30-day cognitive decline. It should be noted that the predictive analysis for the 30-day cognitive decline represents an exploratory extension to a later outcome within an overlapping subset and does not constitute internal or external validation. Trajectory analysis further showed that cognitive outcomes after CEA were heterogeneous, with stable, transient, delayed, and persistent patterns. However, the discharge and 30-day classifications were based on different assessment instruments and thresholds (MoCA decrease ≥ 2 points vs. t-MoCA decrease ≥ 3 points). Therefore, the four trajectory subtypes should be interpreted as approximate classifications based on screening tools rather than precise longitudinal cognitive trajectories.

The definition of cognitive decline warrants further clarification. This threshold was established with reference to previously reported MoCA change cutoffs in relevant cognitive studies, and it is consistent with the literature-reported minimal clinically important difference (MCID) for the MoCA [11]. The minimal detectable change (MDC) of the MoCA in the present study population has not been reported. We attempted a sensitivity analysis using a stricter threshold based on the MDC derived from other populations; however, too few patients met this threshold to permit meaningful analysis. The cognitive decline identified in this study should be interpreted as a screening-detected cognitive change rather than a confirmed neurocognitive disorder based on comprehensive neuropsychological assessment.

Higher preoperative triglyceride levels were independently associated with early cognitive decline in our cohort, although they were not significantly associated with baseline MoCA scores. This suggests that triglycerides may reflect a metabolic-vascular vulnerability that becomes clinically relevant under perioperative hemodynamic stress. Previous studies have linked triglyceride levels to cognitive dysfunction independent of other vascular risk factors, apolipoprotein E4 status, and cerebral white matter microstructure [12,13]. Experimental evidence also suggests that hypertriglyceridemia may impair cognitive function through effects on hippocampal synaptic plasticity [14]. However, clinical evidence remains inconsistent, and some meta-analyses have suggested a possible protective association between higher triglyceride levels and cognitive outcomes [15]. In addition, although preoperative triglyceride levels have been associated with postoperative delirium [16], evidence specific to postoperative cognitive decline after CEA is limited. Therefore, this finding should be regarded as hypothesis-generating and requires validation.

Preoperative cerebral hypoperfusion volume was another independent risk factor, supporting the relevance of baseline brain reserve in postoperative cognitive vulnerability. Hypoperfused tissue may be particularly susceptible to microemboli, ischemia during carotid clamping, and reperfusion-related injury after recanalization [17,18,19]. Chronic cerebral hypoperfusion has also been linked to white matter injury, glial activation, oxidative stress, and cognitive dysfunction in experimental and clinical studies [20,21]. In our study, univariate between-group comparisons in Table 1 showed that the median preoperative hypoperfusion volume was highest in the cognitive improvement group, lowest in the stable cognition group, and intermediate in the cognitive decline group. In crude comparisons, the cognitive decline group did not exhibit higher hypoperfusion volume than the other two groups. After adjusting for confounders in multivariable regression, preoperative hypoperfusion volume was an independent positive predictor of cognitive decline, indicating that greater hypoperfusion volume was associated with an increased risk of postoperative cognitive decline. This discrepancy may arise because univariate between-group comparisons did not account for baseline confounders, and confounding effects masked the true association between hypoperfusion volume and cognitive decline. The predictive effect of hypoperfusion volume on postoperative cognitive decline became evident only after adjustment for relevant confounding variables. And in CEA, the effect of preoperative hypoperfusion is likely bidirectional: revascularization may improve perfusion, but further insufficient perfusion during intraoperative carotid clamping and subsequent rapid reperfusion after clamp release in chronically hypoperfused tissue may also induce transient injury. This may partly explain why hypoperfusion volume was independently associated with cognitive decline, while its effect size was modest.

Surgical laterality was also associated with postoperative cognitive decline. Several studies have reported worse cognitive outcomes in patients with left-sided carotid stenosis or left-sided CEA [5,6,22], which is consistent with our findings. A plausible explanation is hemispheric functional lateralization. Most patients in this cohort were right-handed, and the left hemisphere is typically dominant for language and related cognitive functions in right-handed individuals. Notably, the association between left-sided CEA and postoperative cognitive decline may be partially explained by the inherent characteristics of the MoCA scale rather than purely neurological injury. The MoCA is heavily weighted toward verbal memory, language processing, and verbally mediated executive functions. In this right-handed dominant cohort, left carotid surgery is more likely to induce measurable MoCA reduction caused by transient language functional disturbance, which may reflect testing bias rather than genuine global cognitive deterioration. Furthermore, although handedness exhibited significant intergroup imbalance (*p* = 0.003), it was incorporated into LASSO. Handedness was not retained in the final stable variables. These findings suggest that left-sided CEA is correlated with postoperative cognitive decline rather than confirming a direct causal effect. Given the limited number of left-handed patients and the lack of formal assessment of language dominance in this study, the laterality findings should be interpreted cautiously.

The association between intraoperative MAP decline and cognitive decline underscores the importance of hemodynamic stability during CEA. Patients undergoing CEA often have impaired cerebral autoregulation and heterogeneous autoregulatory thresholds [23]. When blood pressure falls below an individual’s lower autoregulatory limit, cerebral blood flow may decrease, particularly in regions with limited collateral compensation. Intraoperative hypotension and blood pressure variability have been associated with postoperative delirium and other adverse neurological outcomes [24,25,26,27]. It should be noted that the MAP decline in this study was calculated based on intraoperative monitoring data from the entire procedure, representing the maximum decrease relative to baseline, rather than being confined to the post-unclamping phase alone. A previous study has shown that the area under the curve for MAP below a specific threshold is associated with postoperative neurocognitive outcomes [25]. Our findings demonstrate that the maximum intraoperative MAP decrease is also associated with postoperative cognitive decline. Our results suggest that the relative decrease in MAP from baseline may be more informative than the absolute minimum MAP, because it better reflects the magnitude of perfusion stress experienced by each patient. These findings support individualized blood pressure management rather than reliance on uniform MAP thresholds.

VAS on postoperative day 1 was independently associated with early cognitive decline. Pain may contribute to cognitive vulnerability through stress activation, inflammatory mediator release, sleep disruption, and increased sympathetic activity [28,29]. Although evidence linking pain to postoperative delirium is stronger than evidence linking pain to postoperative cognitive decline, inadequate analgesia remains a plausible and modifiable contributor to early postoperative brain dysfunction. These findings support careful postoperative pain assessment and multimodal analgesia in older patients undergoing CEA, while recognizing that causality cannot be inferred from this observational analysis. In particular, early cognitive impairment may alter patients’ subjective perception and self-reporting of pain, potentially affecting the accuracy of VAS scores and giving rise to reverse causation.

Some CEA-specific intraoperative factors were not independently associated with cognitive decline in this cohort. In previous studies, cerebral oxygen desaturation during carotid clamping and longer clamping time have been linked to postoperative cognitive decline [10,30,31,32]. We further explored the potential interaction between ipsilateral rSO_2_ decline during carotid clamping and clamping duration. After mean-centering these two continuous variables, their product interaction term was generated. In our study, neither ipsilateral rSO_2_ decline, clamping time, nor their interaction was associated with the outcome. This may be related to the prophylactic blood pressure augmentation strategy during clamping and relatively short operative times. Notably, the observed nadir rSO_2_ reflects the combined effect of the intrinsic cerebral perfusion disturbance and the patient’s response to hemodynamic treatment. This confounding by indication may limit causal interpretation. In this cohort, no patient received an intraoperative shunt. A previous study has demonstrated that shunt use is not significantly associated with postoperative cognitive changes [33]. However, the standardized intraoperative management protocol in this study, including prophylactic blood pressure augmentation and no use of an intraoperative shunt, may limit the generalizability of the prediction model to different clinical settings. Other factors, such as plaque instability, microembolization, electroencephalographic burst suppression, were not captured in this study and should be incorporated in future risk models [18].

The trajectory analysis provides additional clinical information beyond a single postoperative cognitive assessment. Some patients with early decline recovered by 30 days, suggesting a transient postoperative fluctuation, whereas others developed delayed decline despite normal cognition at discharge. These findings indicate that a discharge assessment alone may miss patients at risk for later deterioration. The early prediction model showed lower discrimination for 30-day decline than for discharge decline, which is expected because home rehabilitation, medication adherence, emotional status, sleep, and other post-discharge factors may influence cognition. Nevertheless, the Sankey diagram highlights that postoperative cognitive change is not uniformly linear and supports structured follow-up for patients with early decline and for high-risk patients who appear stable at discharge.

This study has several strengths. First, cognitive decline was defined using patient-level threshold changes in MoCA or t-MoCA scores rather than mean group differences, which may be more clinically relevant for individualized screening. Second, LASSO-logistic regression was used to select predictors from multidimensional perioperative variables and reduce multicollinearity. Third, cognitive function was assessed at three clinically meaningful time points: before surgery, at discharge, and 30 days after surgery. Several limitations should also be acknowledged. First, although there were no statistically significant differences between patients who completed follow-up and those lost to follow-up, given the limited number of patients lost to follow-up (n = 43), this comparison was underpowered, and the absence of statistically significant differences does not rule out the possibility of selection bias. In addition, the incidence of POD was low and within the range reported previously [23,34], but it may still have been underestimated. POD was retained by LASSO regression but eliminated in the multivariable logistic regression model. Given that only 5 patients developed POD, this variable had insufficient statistical power, and the exclusion of this variable from the final model should not be interpreted as a negative finding. Additionally, this was a single-center study with a modest sample size, and the model showed moderate rather than strong predictive performance and requires external validation. Cognitive assessment relied primarily on MoCA-based screening and did not provide detailed domain-specific neuropsychological profiles. The follow-up period was limited to 30 days, so long-term cognitive outcomes remain unknown. Finally, biomarkers of neurological injury and postoperative imaging indicators, such as diffusion-weighted imaging, were not available; therefore, mechanistic interpretation remains limited.

## 5. Conclusions

Preoperative triglyceride level, cerebral hypoperfusion volume, surgical laterality, intraoperative MAP decline, and VAS on postoperative day 1 were independently associated with early cognitive decline after CEA. A model incorporating these variables showed acceptable discrimination at discharge and moderate discrimination at 30 days. Cognitive trajectories after CEA were heterogeneous, including transient, delayed, and persistent decline. These findings may support perioperative risk stratification and structured postoperative cognitive follow-up. However, no internal or external validation was performed, and further testing is required in larger multicenter cohorts.

## Figures and Tables

**Figure 1:**
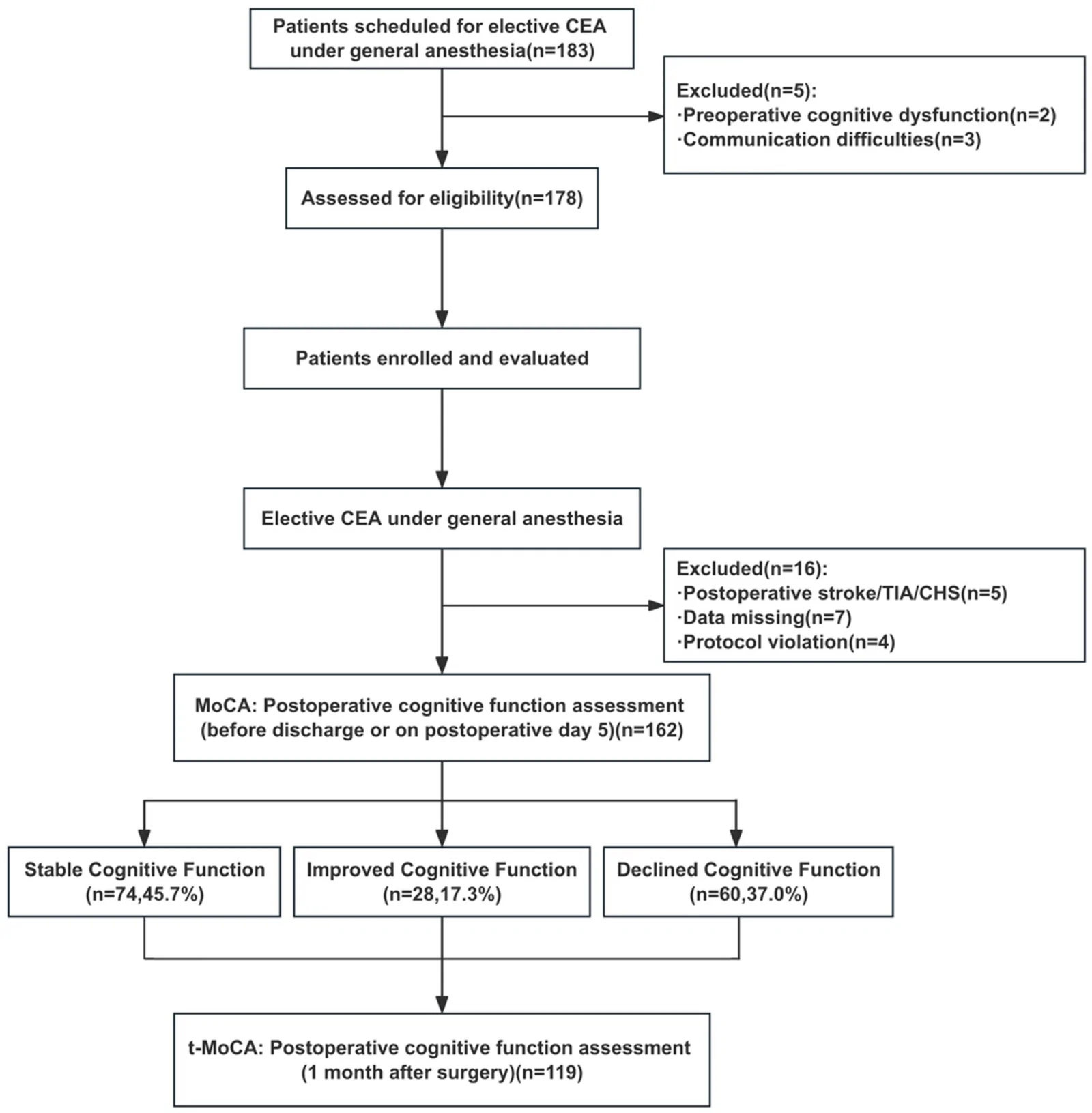
Flowchart of the study.

**Figure 2:**
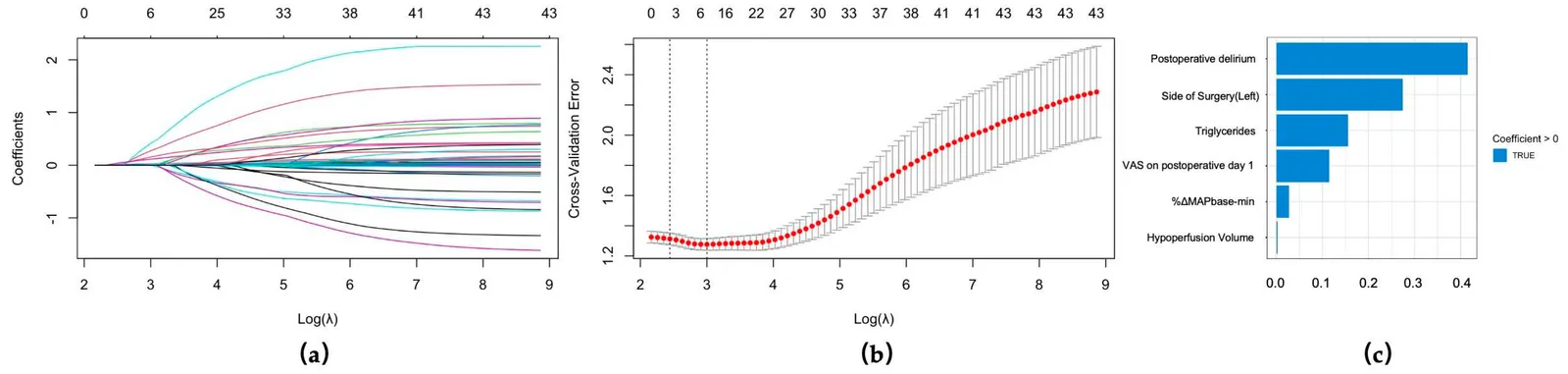
Results of the LASSO regression analysis. (**a**) Plot of the LASSO coefficient profiles; (**b**) Tuning parameter (λ) selection cross-validation error curve; (**c**) Bar plot of regression coefficients.

**Figure 3:**
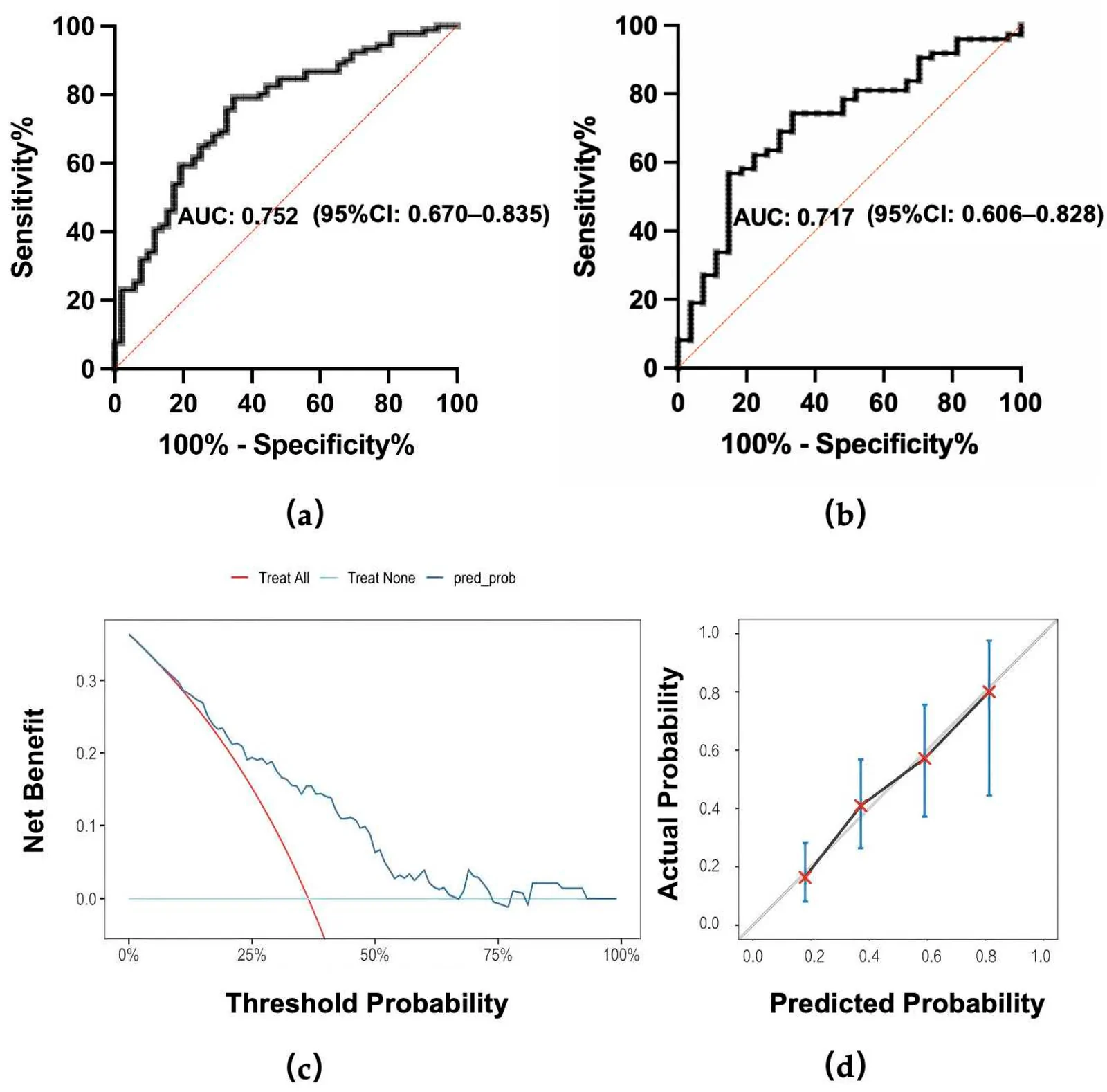
Performance evaluation of the prediction model. (**a**) ROC curve for cognitive decline before discharge; (**b**) ROC curve for cognitive decline at postoperative day 30; (**c**) Decision curve analysis; (**d**) Calibration curve.

**Figure 4:**
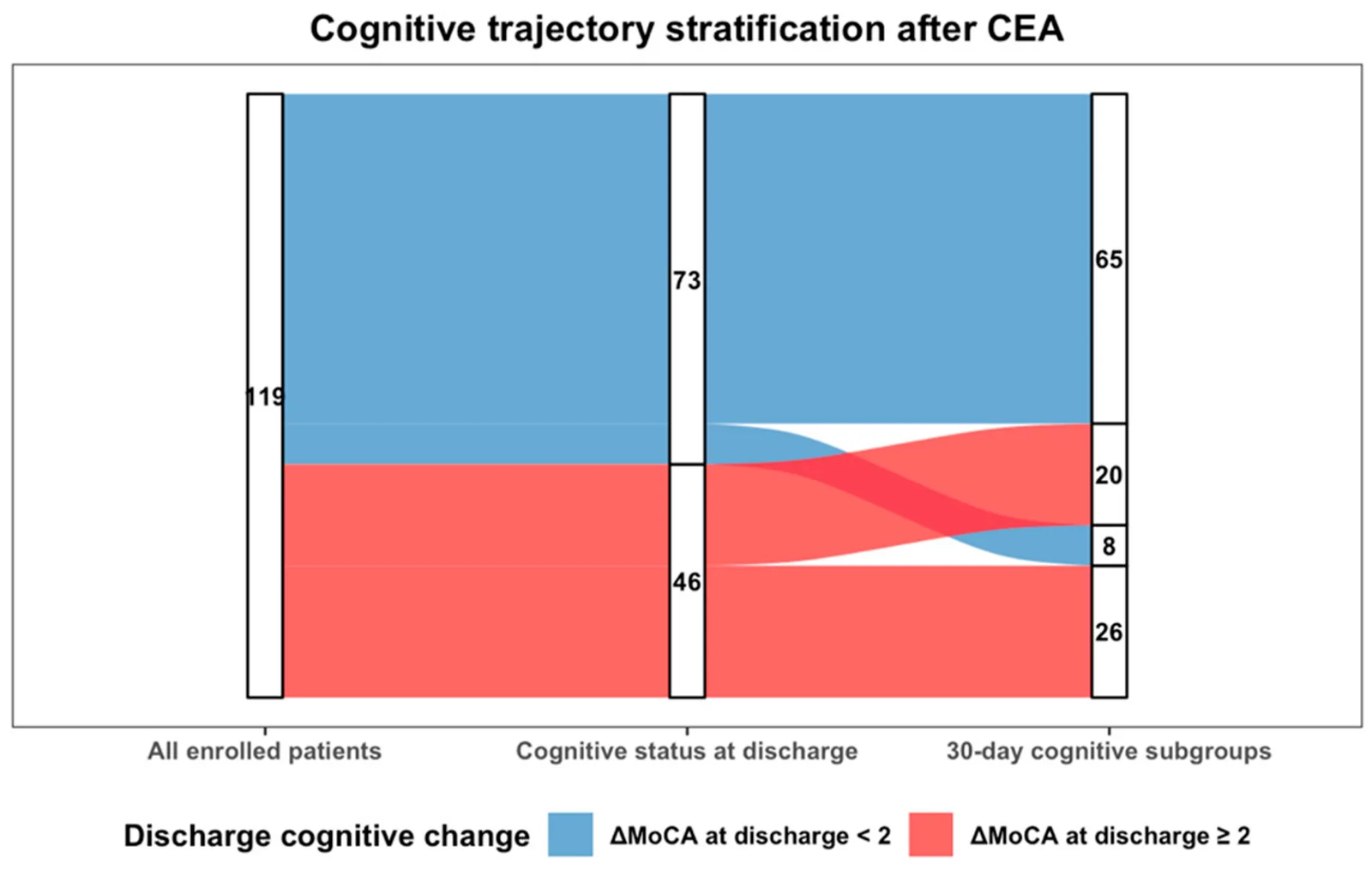
Sankey diagram showing cognitive trajectories after CEA. The left vertical bar represents the 119 patients who completed the 30-day postoperative follow-up. The middle stratum classifies patients by cognitive change at discharge: the blue band indicates MoCA decrease of <2 points (*n* = 73), and the red band indicates MoCA decrease of ≥2 points (*n* = 46). The right strata show 30-day outcomes: stable cognition (*n* = 65), transient cognitive decline (*n* = 20), delayed cognitive decline (*n* = 8), persistent cognitive decline (*n* = 26). The width of each flow band corresponds to the number of patients.

**Table 1:** Comparison of Baseline Demographic and Clinical Characteristics Among Patients Stratified by Cognitive Trajectory at Discharge.

Variables	Stable (*n* = 74)	Improved (*n* = 28)	Declined (*n* = 60)	*p*-Value
Preoperative Data				
Age (Q1–Q3)	68 (64–72)	62 (61–69)	68 (62–71)	0.018 *
Sex (Male%)	59 (79.7%)	25 (89.3%)	44 (73.3%)	0.375
BMI (Mean ± SD)	24.031 (2.733)	25.454 (2.499)	25.144 (2.825)	0.021 *
Smoking History (%)	46 (62.2%)	14 (50.0%)	32 (53.3%)	0.461
Alcohol Consumption History (%)	35 (47.3%)	13 (46.4%)	19 (31.7%)	0.183
Dominant Hand (Left%)	3 (4.1%)	6 (21.4%)	2 (3.3%)	0.003 **
≤Primary School (%)	14 (18.9%)	3 (10.7%)	12 (20.0%)	0.571
Physical Exercise (%)	28 (37.8%)	10 (35.7%)	25 (41.7%)	0.826
Recreational Activities (%)	27 (36.5%)	6 (21.4%)	15 (25.0%)	0.188
Hypertension (%)	40 (54.1%)	16 (57.1%)	37 (61.7%)	0.651
Coronary Artery Disease (%)	23 (31.1%)	10 (35.7%)	23 (38.3%)	0.664
Diabetes (%)	18 (24.3%)	11 (39.3%)	21 (35.0%)	0.221
Cerebral Infarction (%)	22 (29.7%)	12 (42.9%)	19 (31.7%)	0.419
Respiratory Dysfunction (%)	22 (29.7%)	5 (17.9%)	11 (18.3%)	0.272
Hypoperfusion Volume (Q1–Q3)	1.100 (0.000–4.210)	9.200(2.980–37.640)	2.390 (0.370–16.060)	0.210
Infarct Core Volume (Q1–Q3)	0.000 (0.000–0.390)	0.280 (0.000–2.980)	0.035 (0.000–0.800)	0.424
White Blood Cell (Q1–Q3)	6.260 (5.620–7.025)	6.420 (5.040–6.610)	5.990 (5.230–6.660)	0.833
Red Blood Cell (Mean ± SD)	4.627 (0.508)	4.686 (0.423)	4.589 (0.547)	0.711
Hemoglobin (Mean ± SD)	143.69 (13.84)	143.29 (15.23)	141.57 (16.00)	0.709
Total Cholesterol (Q1–Q3)	3.330 (2.865–4.340)	3.890 (3.410–4.430)	3.555 (3.130–4.070)	0.561
Triglycerides (Q1–Q3)	1.170 (0.820–1.570)	1.180 (0.950–1.420)	1.205 (0.940–1.670)	0.150
High-Density Lipoprotein (Q1–Q3)	1.030 (0.935–1.285)	1.050 (0.950–1.290)	1.155 (0.940–1.270)	0.922
Low-Density Lipoprotein (Q1–Q3)	1.880 (1.525–2.350)	2.150 (1.990–2.840)	2.015 (1.540–2.280)	0.576
Baseline MAP (Mean ± SD)	93.019 (10.987)	94.358 (12.254)	97.994 (12.049)	0.053
PSQI (Q1–Q3)	4 (3–6)	3 (3–6)	4 (3–7)	0.818
SAS (Q1–Q3)	32.00 (30.00–36.50)	32.00 (30.00–35.00)	33.00 (31.00–36.00)	0.566
Baseline MoCA (Q1–Q3)	24 (21–26)	24 (22–26)	25 (23–27)	0.074
Intraoperative Data				
Side of Surgery (Left%)	26 (35.1%)	9 (32.1%)	28 (46.7%)	0.284
%Ipsilateral rSO_2_ decline*Clamp time (Q1–Q3)	−0.036 (−0.623–0.103)	−0.098 (−1.611–0.011)	−0.127 (−0.454–0.111)	0.434
%ΔMAPmax-base (Mean ± SD)	0.167 (0.128)	0.148 (0.113)	0.112 (0.137)	0.064
%ΔMAPbase-min (Mean ± SD)	0.212 (0.113)	0.249 (0.104)	0.273 (0.103)	0.009 **
%ΔMAPmax-min (Mean ± SD)	0.349 (0.088)	0.373 (0.083)	0.361 (0.087)	0.462
Dose of Sufentanil (Q1–Q3)	20.00 (15.00–20.00)	20.00 (15.00–20.00)	17.50 (15.00–20.00)	0.776
Anesthesia Time (Q1–Q3)	113 (95–122)	110 (96–122)	111 (91–127)	0.713
Postoperative Data				
VAS on postoperative day 1 (Q1–Q3)	3 (2–3)	3 (2–3)	4 (2–5)	0.056
POD (%)	1 (1.4%)	0 (0.0%)	4 (6.7%)	0.122

Significant at * *p* < 0.05, ** *p* < 0.01; Q1: first quartile; Q3: third quartile; SD: standard deviation; PSQI: Pittsburgh Sleep Quality Index; SAS: Self-Rating Anxiety Scale; %Ipsilateral rSO_2_ decline = [(baseline ipsilateral rSO_2_ − nadir ipsilateral rSO_2_)/baseline ipsilateral rSO_2_] × 100%; MoCA: Montreal Cognitive Assessment; POD: postoperative delirium; VAS: visual analogue scale; MAP: mean arterial pressure; %ΔMAPmax-base = [(peak MAP − baseline MAP)/peak MAP] × 100%; %ΔMAPbase-min = [(baseline MAP − nadir MAP)/baseline MAP] × 100%; %ΔMAPmax-min = [(peak MAP − nadir MAP)/peak MAP] × 100%.

**Table 2:** Multivariate logistic regression analysis.

Variable	B	Standard Error	Wald χ2	*p* Value	OR	95%CI
Triglycerides (mmol/L)	0.613	0.269	5.194	0.023 *	1.846	1.090–3.127
Hypoperfusion Volume(mL)	0.008	0.004	3.993	0.046 *	1.008	1.000–1.017
Side of Surgery (Left)	0.920	0.402	5.234	0.022 *	2.510	1.141–5.523
%ΔMAPbase-min	0.047	0.018	6.676	0.010 *	1.049	1.012–1.087
VAS on postoperative day 1	0.280	0.103	7.332	0.007 **	1.323	1.080–1.621

Significant at * *p* < 0.05, ** *p* < 0.01; %ΔMAPbase-min = [(baseline MAP − nadir MAP)/baseline MAP] × 100%, entered as a continuous variable per 1-percentage-point increase in multivariable logistic regression; VAS: visual analogue scale.

## Data Availability

The datasets generated and analyzed in this prospective observational study cannot be publicly released due to restrictions imposed by ethical regulations and patient privacy protection. Qualified researchers may submit reasonable applications for de-identified clinical data.

## Supplementary Materials

The following supporting information can be downloaded at: https://www.mdpi.com/article/10.3390/brainsci16101032/s1, Table S1: Bootstrap selection frequencies of the six retained variables (1000 iterations); Table S2: Multivariable logistic regression analysis with baseline MoCA adjustment; Table S3: Comparison of Baseline Demographic and Clinical Characteristics Between Patients With and Without 30-Day Follow-up.

## Abbreviations

The following abbreviations are used in this manuscript:

CEA	Carotid endarterectomy
MoCA	Montreal cognitive assessment
CTP	Computed tomography perfusion
MAP	Mean arterial pressure
VAS	Visual analogue scale
AUC	Area under the curve
ASA	American society of anesthesiologists
ADL	Activities of daily living
BMI	Body mass index
PSQI	Pittsburgh sleep quality index
SAS	Self-rating anxiety scale
rSO2	Regional cerebral oxygen saturation
3D-CAM	3-Minute diagnostic interview for confusion assessment method
LASSO	Least absolute shrinkage and selection operator
ORs	Odds ratios
TIA	Transient ischemic attack
CHS	Cerebral hyperperfusion syndrome
CIs	Confidence intervals
ROC	Receiver operating characteristic
MCID	Minimal clinically important difference
MDC	Minimal detectable change
