# Supplementary material for: Perioperative Predictors and Temporal Trajectories of Postoperative Cognitive Decline After Carotid Endarterectomy: A Prospective Cohort Study
Source: Brain Sci. 2026 Sep 27;16(10):1032. doi: 10.3390/brainsci16101032 (PMC13702362; doi:10.3390/brainsci16101032)
Supplement: Supplementary file 1 [file brainsci-16-01032-s001.zip › brainsci-4491064-supplementary.pdf]

Table S1 Bootstrap selection frequencies of the six retained variables (1000 iterations)

Rank	Variable	Selection frequency
1	%ΔMAPbase－min	0.859
2	VAS on postoperative day 1	0.809
3	Side of Surgery(Left)	0.662
4	Triglycerides	0.562
5	Hypoperfusion Volume	0.559
6	Postoperative Delirium	0.531

%ΔMAPbase－min = [(baseline MAP－nadir MAP)/baseline MAP] × 100%, entered as a continuous variable per 1-percentage-point increase in multivariable logistic regression; VAS: visual analogue scale. Bootstrap resampling was performed with 1000 iterations. In each iteration, LASSO regression was re-run, and the selection frequency of each candidate variable was recorded. The six retained variables all had selection frequencies greater than 0.5. All other candidate variables had selection frequencies below 0.5

Table S2 Multivariable logistic regression analysis with baseline MoCA adjustment

Variable	B	Standard error	Wald $\chi 2$	P value	OR	95%CI
Triglycerides	0.669	0.262	6.525	0.011*	1.952	1.168–3.262
Hypoperfusion Volume	0.009	0.004	4.999	0.025*	1.010	1.001–1.018
Side of Surgery(Left)	0.961	0.412	5.435	0.020*	2.613	1.165–5.860
%ΔMAPbase－min	0.053	0.019	7.534	0.006**	1.055	1.015–1.095
VAS on postoperative day 1	0.292	0.105	7.713	0.005**	1.340	1.090–1.647
Baseline MoCA	0.151	0.067	4.985	0.026*	1.163	1.019–1.327

Significant at *p<0.05, **p<0.01; %ΔMAPbase－min = [(baseline MAP－nadir MAP)/baseline MAP] × 100%, entered as a continuous variable per 1-percentage-point increase in multivariable logistic regression; VAS: visual analogue scale; MoCA: Montreal Cognitive Assessment

Table S3. Comparison of Baseline Demographic and Clinical Characteristics Between Patients With and Without 30-Day Follow-up

**Variables**	**Completed Follow-up(*n* = 119)**	**Lost to Follow-up(*n* = 43)**	***p*-Value**
Preoperative Data			
Age(Q1–Q3)	66(62–71)	69(63–73)	0.119
Sex(Male%)	92(77.3%)	36(83.7%)	0.605
BMI(Mean ± SD)	24.753(2.774)	24.146(3.068)	0.241
Smoking History(%)	70(59.3%)	23(53.5%)	0.589
Alcohol Consumption History(%)	50(42.4%)	16(37.2%)	0.591
Dominant Hand(Left%)	6(5.1%)	5(11.6%)	0.165
≤ Primary School(%)	17(14.5%)	12(27.9%)	0.065
Physical Exercise(%)	45(38.5%)	18(41.9%)	0.817
Recreational Activities(%)	33(28.4%)	14(32.6%)	0.696
Hypertension(%)	70(63.1%)	24(60.0%)	0.849
Coronary Artery Disease(%)	39(35.1%)	18(45.0%)	0.342
Diabetes(%)	40(36.0%)	9(22.5%)	0.167
Cerebral Infarction(%)	39(35.1%)	13(32.5%)	0.847
Respiratory Dysfunction(%)	27(25.5%)	12(35.3%)	0.279
Hypoperfusion Volume(Q1–Q3)	1.900(0.000–11.773)	1.640(0.630–8.500)	0.729
Infarct Core Volume(Q1–Q3)	0.000(0.000–0.475)	0.000(0.000–0.225)	0.928
White Blood Cell(Q1–Q3)	6.130(5.395–7.160)	6.290(5.360–6.940)	0.890
Red Blood Cell(Mean ± SD)	4.645(0.496)	4.547(0.525)	0.274
Hemoglobin(Mean ± SD)	143.000(14.671)	141.420(15.027)	0.549
Total Cholesterol(Q1–Q3)	3.580(2.963–4.248)	3.630(3.000–4.380)	0.506
Triglycerides(Q1–Q3)	1.140(0.870–1.530)	1.180(0.900–1.560)	0.707
High-Density Lipoprotein(Q1–Q3)	1.090(0.970–1.278)	1.070(0.870–1.250)	0.235
Low-Density Lipoprotein(Q1–Q3)	1.925(1.470–2.275)	2.030(1.600–2.660)	0.163
Baseline MAP(Mean ± SD)	95.257(11.938)	95.240(11.798)	0.994
PSQI(Q1–Q3)	4(3–6)	5(3–7)	0.670
SAS(Q1–Q3)	33.00(31.00–36.25)	32.00(30.00–36.00)	0.596
Baseline MoCA(Q1–Q3)	25(21–27)	24(22–26)	0.303
Intraoperative Data			
Side of Surgery(Left%)	45(39.8%)	19(47.5%)	0.457
%Ipsilateral rSO2 decline*Clamp time(Q1–Q3)	-0.080(-0.498–0.096)	-.0148(-1.213–0.423)	0.577
%ΔMAPmax－base(Mean ± SD)	0.137(0.128)	0.156(0.140)	0.437
%ΔMAPbase－min(Mean ± SD)	0.238(0.110)	0.249(0.116)	0.602
%ΔMAPmax－-min(Mean ± SD)	0.351(0.086)	0.375(0.086)	0.131
Anesthesia Time(Q1–Q3)	112(94–125)	105(83–124)	0.256
Postoperative Data			

VAS on postoperative day 1(Q1−Q3)	3.00(2.00–5.00)	3.00(2.00–4.75)	0.373
POD(%)	5(4.2%)	0(0.0%)	0.326
Postoperative MoCA(Q1−Q3)	23(21−26)	24(21−26)	0.790
Declined Postoperative Cognitive(%)	46(38.7%)	14(32.6%)	0.581

Significant at *p<0.05, **p<0.01; Q1: First Quartile; Q3: Third Quartile; SD: Standard Deviation; PSQI: Pittsburgh Sleep Quality Index; SAS: Self-Rating Anxiety Scale; %Ipsilateral rSO2 decline= [(Baseline ipsilateral rSO − Nadir ipsilateral rSO_2)/ Baseline ipsilateral rSO_2] × 100%; MoCA: Montreal Cognitive Assessment; POD: Postoperative Delirium; VAS: Visual Analogue Scale; MAP: Mean arterial pressure; %ΔMAPmax − base=[(Peak MAP − Baseline MAP)/Peak MAP]×100%; %ΔMAPbase − min=[(Baseline MAP − Nadir MAP)/Baseline MAP]× 100%; %ΔMAPmax − min=[(Peak MAP − Nadir MAP) / Peak MAP] × 100%
